# Neodactylariales, Neodactylariaceae (Dothideomycetes, Ascomycota): new order and family, with a new species from China

**DOI:** 10.3897/mycokeys.73.54054

**Published:** 2020-09-11

**Authors:** Min Qiao, Hua Zheng, Ruili Lv, Zefen Yu

**Affiliations:** 1 Laboratory for Conservation and Utilization of Bio-resources, Key Laboratory for Microbial Resources of the Ministry of Education, Yunnan University, Kunming, Yunnan, 650091, China Yunnan University Kunming China

**Keywords:** Dothideomycetes, new family, new order, new species, phylogenetic analysis, taxonomy

## Abstract

During a mycological survey of aquatic hyphomycetes on submerged decaying leaves in southwest China, a distinct new fungus was collected. The collection was cultured and sequenced and a BLAST search of its ITS and LSU sequence against data in GenBank revealed a dothideomycetous affiliation, with the closest related taxa in the genus *Neodactylaria*. Phylogenetic analyses of a multigene matrix containing sequences from four genes (LSU, SSU, *rpb*2, and *tef*1), representing broad groups of Dothideomycetes, revealed its placement within Dothideomycetes, but without a supported familial or ordinal affiliation. Based on further phylogenetic analyses and morphological investigations, the new fungus is described here as a new species of *Neodactylaria*, *N. simaoensis***sp. nov.**, and placed in a new family Neodactylariaceae**fam. nov.** and a new order Neodactylariales**ord. nov.**

## Introduction

The kingdom Fungi contains an estimated 700,000 to over 5 million species, amongst which only about 120,000 have been described (Lynne 2016). Dothideomycetes is one of the largest and most significant classes of fungi within Ascomycota (Kirk et al. 2008; Schoch et al. 2009a; Hyde et al. 2013). Thousands of species have been included in the class Dothideomycetes, and many of them are important plant pathogens (Cortinas et al. 2006; Crous et al. 2007; Wikee et al. 2011, 2013a, b; Manamgoda et al. 2012), human and animal pathogens (Siu and Lzumi 2004; da Cunha et al. 2012, 2013), or used in biotechnological applications (Verkley et al. 2004; Damm et al. 2008; de Wit et al. 2012; Ohm et al. 2012; Stergiopoulos et al. 2012; Hyde et al. 2014). The members of Dothideomycetes are still increasing with the discovery of many novel species and inclusion of DNA sequence data. In the past few years, molecular phylogenetic studies have advanced our understanding of the systematics of Dothideomycetes (Inderbitzin et al. 2001; Schoch et al. 2009b; Hirayama et al. 2010; Suetrong et al. 2011; Hyde et al. 2013; Wijayawardene et al. 2014; Liu et al. 2017; Jiang et al. 2020). Wijayawardene et al. (2014) recommended 23 orders and 110 families in Dothideomycetes based on culture characteristics and molecular phylogenetic analyses. More recently, Liu et al. (2017) provided an updated phylogenetic assessment of Dothideomycetes at the order level by using molecular clock methods and accepted 29 orders. However, the latest research by Wijayawardene et al. (2018) expanded this to 33. Despite the progress in our understanding of the systematics of Dothideomycetes, a number of newly described and/or previously reported taxa are currently *incertae sedis* and their family and order level positions within the Dothideomycetes remain obscure; many taxa lack sequencing data or appropriate classification rank to accommodate them (Hyde et al. 2013; Wijayawardene et al. 2018).

The genus *Neodactylaria* Guevara-Suarez et al., typified by *N. obpyriformis* Guevara-Suarez et al., was originally described from human bronchoalveolar lavage in the USA (Crous et al. 2017). The genus is characterized by having integrated, polyblastic and sympodial extended conidiogenous cells producing solitary, septate, obpyriform or rostrate conidia (Crous et al. 2017). Morphologically, *Neodactylaria* is similar to two *Dactylaria* species, *D. kumamotoensis* Matsush. and *D. madrasensis* Matsush., and several *Pyricularia* species, such as *P. grisea* Cooke ex Sacc. and *P. pennisetigena* Klaubauf, M.-H. Lebrun & Crous. However, in the phylogeny inferred from sequences of the large subunits of nuclear ribosomal DNA (LSU), *Neodactylaria* was placed within Dothideomycetes, but the ordinal and familial position was unresolved.

Southwestern China is one of the world’s 34 biodiversity hotspots (Myers et al. 2000; Zhang et al. 2020). During a survey of aquatic hyphomycetes on submerged decaying leaves from this area, several new species have been reported (Guo et al. 2019; Qiao et al. 2019a, b; Yu et al. 2019). In a further study, an unidentified fungus was collected, which had a similar morphology to *Heliocephala proliferans* V. Rao et al. (Pezizomycotina*incertae sedis*; Rao et al. 1984; Mel’nik et al. 2013), but detailed morphological examination showed that the conidiogenous cells were terminal or intercalary, with short-cylindrical denticles, and the conidia were 1- or 2-septate and constricted at the septum. Sequence data obtained from cultures of conidia confirmed that this species does not belong in *Heliocephala*. A BLAST search of its LSU gene sequences against the public sequence records in GenBank (Sayers et al. 2019) confirmed its dothideomycetous affinity and that it was closely related to members of the genus *Neodactylaria*. Subsequently, we obtained the type species of *Neodactylaria*, *N. obpyriformis* Guevara-Suarez et al., from the CBS-KNAW Fungal Biodiversity Centre (Netherlands) and processed it with full morphological and phylogenetic analyses. Our new collection prompted the study of the molecular phylogenetic relationships of taxa within *Neodactylaria*, as well as the higher order phylogenetic relationship of *Neodactylaria* within the Dothideomycetes.

Our comparative analyses identified that the newly collected fungus is a species of *Neodactylaria*, *N. simaoensis*. However, due to their significant divergence, there was no apparent family or order for placement of *Neodactylaria*. We propose that the genus be placed in a new family and new order within Dothideomycetes.

## Materials and methods

### Isolation and morphological study

Submerged dicotyledonous leaves were collected from a stream in Simao, Yunnan Province, southern China. Samples were preserved in zip-lock plastic bags, labelled and transported to the laboratory. Each rotted leaf was cut into several 3–4 × 4–5 cm sized fragments, and these were incubated on CMA (20 g cornmeal, 18 g agar, 1000 ml distilled water), supplemented by two antibiotics (penicillin G, 0.04 g/l; and streptomycin, 0.03 g/l; Gams et al. 1998), for 5 days at room temperature. Individual conidia were isolated using a sterilised toothpick under a BX51 microscope and cultivated on CMA plates. Morphological observations were conducted on cultures growing on CMA after incubation at 25 °C for 1 week. Colony colour was based on the colour charts of Rayner (1970).

Pure cultures have been deposited in the Herbarium of the Laboratory for Conservation and Utilization of Bio-resources, Yunnan University, Kunming, Yunnan, P.R. China (YMF, formerly Key Laboratory of Industrial Microbiology and Fermentation Technology of Yunnan).

### DNA extraction, polymerase chain reaction (PCR) amplification and sequencing

Pure cultures were grown on PDA for 5 days at 25 °C. Actively-growing mycelia were scraped off the surface of a culture and transferred to 2 ml Eppendorf micro-centrifuge tubes. Total genomic DNA was extracted according to the procedures in Turner et al. (1997). To determine the phylogenetic position of *Neodactylaria*, we amplified five nuclear genomic loci, including the internal transcribed spacer (ITS), the 28S large subunit ribosomal RNA (LSU), the 18S small subunit ribosomal RNA (SSU), the translation elongation factor1-alpha partial gene (*tef*1) and the RNA polymerase II subunit 2 (*rpb*2). The following primers were used: the ITS region was amplified using the primers ITS1 and ITS4 (White et al. 1990); the LSU nuc rDNA region was amplified with primers LROR and LR7 (Vilgalys and Hester 1990); the SSU nuc rDNA region was amplified with primers NS1 and NS4 (White et al. 1990); an approx. 1.1 kb fragment of the *rpb*2 gene was amplified using the primer pair fRPB2-5f and fRPB2-7cr (Liu et al. 1999); an approximately 1.0 kb fragment of the *tef*1 gene was amplified with the primers TEF983F and TEF2218R (initially obtained from S. Rehner: http://ocid.nacse.org/research/deephyphae/EF1primer.pdf).

PCR reactions were prepared in a 25 μl final volume as described by Zheng et al. (2019, 2020a). PCR amplifications were performed in an Eppendorf Mastercycler thermal cycler. PCR conditions were as follows: an initial 4 min denaturing step at 94 °C, followed by 35 cycles of 75 s at 94 °C, 90 s at 52 °C (for *rpb*2, LSU, and SSU) and 100 s at 72 °C. After a final extension step of 7 min at 72 °C, the samples were stored at 4 °C. Conditions for amplification of the ITS and *tef*1 regions were an initial step of three cycles at an annealing temperature of 54 °C, followed by 30 cycles with the annealing temperature set at 48 °C. When needed, a ‘touchdown’ (Don et al. 1991) protocol preceded the PCR cycle. PCR products were then purified using a commercial kit (Bioteke Biotechnology Co. Ltd, China). Each fragment was sequenced from both directions using the forward and reverse primers in separate reactions using a LI-COR 4000L automatic sequencer as described by Kindermann et al. (1998). The sequences obtained have been submitted to GenBank at the National Center for Biotechnology Information (NCBI) and the accession numbers are listed in Table 1.

**Table 1.** Species, strains, and their corresponding GenBank accession numbers of sequences used for phylogenetic analyses.

Species	Strain^a,b^	GenBank accession numbers^c^		
		LSU	SSU	*tef*1
*Acanthostigma chiangmaiense* Boonmee & K.D. Hyde	MFLUCC 10-0125^T^	JN865197	JN865185	KF301560
*Allophaeosphaeria muriformis* Ariyaw., Camporesi & K.D. Hyde	MFLUCC 13-0349^T^	KP765681	KP765682	–
*Bambusaria bambusae* (J.N. Kapoor & H.S. Gill) Jaklitsch, D.Q. Dai, K.D. Hyde and Voglmayr	CBS 139763	KP687813	KP687962	KP687983
*Botryobambusa fusicoccum* Phook., Jian K. Liu & K.D. Hyde	MFLUCC 11-0143^T^	JX646809	JX646826	–
*Botryosphaeria agaves* (Henn.) E.J. Butler	MFLUCC 11-0125^T^	JX646808	JX646825	–
*Botryosphaeria dothidea* (Moug.) Ces. & De Not.	CBS 115476	DQ678051	DQ677998	DQ767637
*Cophinforma atrovirens* (Mehl & Slippers) A. Alves & A.J.L. Phillips	MFLUCC 11-0425^T^	JX646817	JX646833	–
*Dematiopleospora mariae* Wanas., Camporesi, E.B.G. Jones & K.D. Hyde	MFLUCC 13-0612^T^	KJ749653	KJ749652	KJ749655
*Dothidea hippophaes* Fuckel	CBS 188.58	DQ678048	U42475	DQ677887
*Dothidea insculpta* Wallr.	CBS 189.58	DQ247802	DQ247810	DQ471081
*Gloniopsis praelonga* (Schwein.) Underw. & Earle	CBS 112415	FJ161173	FJ161134	FJ161090
*Helicangiospora lignicola* Boonmee, Bhat & K.D. Hyde	MFLUCC 11-0378^T^	KF301531	KF301539	KF301552
*Helicoma chiangraiense* Boonmee & K.D. Hyde	MFLUCC 10-0115	JN865188	JN865176	KF301551
*Helicoma fagacearum* Boonmee & K.D. Hyde	MFLUCC 11-0379	KF301532	KF301540	KF301553
*Hysterium angustatum* Alb. & Schwein.	CBS 236.34	FJ161180	GU397359	FJ161096
*Hysterobrevium smilacis* (Schwein.) E. Boehm & C.L. Schoch	CBS 114601	FJ161174	FJ161135	FJ161091
*Hysteropatella clavispora* (Peck) Höhn.	CBS 247.34	AY541493	DQ678006	DQ677901
*Kellermania macrospora* (Durieu & Mont.) Minnis & A.H. Kenn.	CBS 131716^T^	JX444874	JX444902	–
*Kellermania yuccigena* Ellis & Everh.	CBS 131727	JX444883	JX444908	–
*Minutisphaera aspera* Raja, Oberlies, Shearer & A.N. Mill.	DSM 29478^T^	KP309993	KP309999	–
*Minutisphaera fimbriatispora* Shearer, A.N. Mill. & A. Ferrer	A242-8a	HM196367	HM196374	–
*Minutisphaera japonica* Kaz. Tanaka, Raja & Shearer	JCM 18560^T^	AB733440	AB733434	–
*Murispora rubicunda* (Niessl) Y. Zhang ter, J. Fourn. & K.D. Hyde	IFRD 2017	FJ795507	GU456308	GU456289
*Myriangium duriaei* Mont. & Berk.	CBS 260.36	DQ678059	AY016347	DQ677900
*Myrmaecium rubrum* (Aptroot, Aa & Petrini) Jaklitsch & Voglmayr	CBS 109505	GU456324	GU456303	GU456260
*Myrmaecium fulvopruinatum* (Berk.) Jaklitsch & Voglmayr	CBS 139058	KP687861	KP687968	KP688030
*Myrmaecium rubricosum* (Fr.) Fuckel	CBS 139068	KP687885	KP687979	KP688053
*Neodactylaria obpyriformis* Guevara-Suarez, Deanna A. Sutton, Wiederh. & Gené	CBS 142668	**MK562751**	**MK562750**	–
*Neodactylaria simaoensis* H. Zheng & Z.F. Yu	YMF 1.3984	**MH379210**	**MK562747**	**MK562748**
*Oedohysterium insidens* (Schwein.) E. Boehm & C.L. Schoch	CBS 238.34	FJ161182	FJ161142	FJ161097
*Parawiesneriomyces syzygii* Crous & M.J. Wingf.	CBS 141333^T^	KX228339	–	–
*Patellaria atrata* (Hedw.) Fr.	CBS 958.97	GU301855	GU296181	GU349038
*Phaeotrichum benjaminii* Malloch & Cain	CBS 541.72	AY004340	AY016348	DQ677892
*Phyllosticta ampelicida* (Engelm.) Aa	CBS 237.48	DQ678085	DQ678034	–
*Phyllosticta citricarpa* (McAlpine) Aa	CBS 102374	GU301815	GU296151	GU349053
*Populocrescentia forlicesenensis* Wanas., Camporesi, E.B.G. Jones & K.D. Hyde	MFLUCC 14-0651^T^	KT306952	KT306955	–
*Pseudogliophragma indica* Phadke & V.G. Rao	MTCC 11985^T^	KM052851	KM052852	–
*Psiloglonium araucanum* (Speg.) E. Boehm, Marinc. & C.L. Schoch	CBS 112412	FJ161172	FJ161133	FJ161089
*Saccharata proteae* (Wakef.) Denman & Crous	CBS 115206	GU301869	GU296194	GU349030
*Schismatomma decolorans* (Erichsen) Clauzade & Vězda	DUKE 47570	AY548815	AY548809	DQ883725
*Speiropsis pedatospora* Tubaki	CBS 397.59	KR869797	–	–
*Trematosphaeria pertusa* Fuckel	CBS 122368	FJ201990	FJ201991	GU456276
*Trematosphaeria pertusa* Fuckel	CBS 122371	FJ201992	FJ201993	GU349085
*Trichodelitschia bisporula* (P. Crouan & H. Crouan) Munk	CBS 262.69	GU348996	GU349000	GU349020
*Trichodelitschia munkii* N. Lundq.	Kruys 201	DQ384096	DQ384070	–
*Tubeufia chiangmaiensis* Boonmee & K.D. Hyde	MFLUCC 11-0514^T^	KF301538	KF301543	KF301557
*Tubeufia javanica* Penz. & Sacc.	MFLUCC 12-0545^T^	KJ880036	KJ880035	KJ880037
*Valsaria insitiva* (Tode) Ces. & De Not.	CBS 127882^T^	KP687886	KP687980	KP688054
*Valsaria lopadostomoides* Jaklitsch & Voglmayr	CBS 139062^T^	KP687868	KP687972	KP688037
*Valsaria neotropica* Jaklitsch, J. Fourn. & Voglmayr	CBS 139064^T^	KP687874	KP687974	KP688042
*Valsaria robiniae* (Schwein.) Cooke	CBS 139063	KP687870	KP687973	KP688039
*Valsaria rudis* (P. Karst. & Har.) Theiss. & Syd. ex Petr. & Syd.	CBS 139066^T^	KP687879	KP687976	KP688047
*Valsaria spartii* Maubl.	CBS 139070^T^	KP687843	KP687964	KP688013

^a^ ex-type strains are indicated with ^T^ after the strain number. ^b^ Abbreviations of culture collections (where known): CBS, Westerdijk Fungal Biodiversity Institute, Utrecht, The Netherlands; DSMZ, German Collection of Microorganisms and Cell Cultures, Braunschweig, Germany; G, University of North Carolina, Greensboro, Department of Chemistry and Biochemistry Fungal Culture Collection; DUKE, Duke University Herbarium, Durham, North Carolina; IFRDCC, International Fungal Research and Development Culture Collection; JCM, Japan Collection of Microorganism, RIKEN BioResource Center, Japan; MFLUCC, Japan Collection of Microorganism, RIKEN BioResource Center, Japan; YMF, the Herbarium of the Laboratory for Conservation and Utilization of Bio-resources, Yunnan University, China. ^c^ Sequences obtained in this study are shown in bold.

### Sequence alignment and phylogenetic analysis

Preliminary BLAST searches with ITS, SSU, LSU, *rpb*2, and *tef*1 gene sequences of the new isolate against GenBank and UNITE databases (Nilsson et al. 2019) identified sequences closely related to our isolates. However, we were only able to robustly determine their placements within the class Dothideomycetes. To infer a phylogenetic relationship for our strain, an initial alignment of the newly generated sequences (SSU, LSU, *rpb*2, and *tef*1) and 74 representatives belonging to 33 orders of the Dothideomycetes, extracted from recent studies (Mapook et al. 2016; Nieuwenhuijzen et al. 2016; Voglmayr et al. 2016; Hernandez-Restrepo et al. 2017; Liu et al. 2017; Wijayawardene et al. 2018) with a species from the sibling class, Arthoniomycetes, as the outgroup, was performed using the online MAFFT interface (Katoh and Standley 2013; http://mafft.cbrc.jp/alignment/server). This alignment was used to infer a preliminary phylogenetic relationship for the new sequences based on Bayesian inference (BI) analyses (data not shown).

Based on the initial analysis, a second alignment combined SSU, LSU, and *tef*1 sequence data were constructed from the closest relatives to our strain in Botryosphaeriales, Dothideales, Hysteriales, Minutisphaerales, Myriangiales, Patellariales, Phaeotrichales, Pleosporales, Tubeufiales, and Venturiales. In the second alignment, *Schismatomma decolorans* (DUKE 47570) was used as an outgroup taxon. All sequence data were aligned using MAFFT (v. 7.110) online program (http://mafft.cbrc.jp/alignment/server/) (Katoh and Standley 2013). The alignments were checked and uninformative gaps minimized manually where necessary in BioEdit 7.0.1 (Hall 1999). Maximum likelihood (ML) and BI were used in the analyses following the methodology as described in Mapook et al. (2016). The nucleotide substitution models use for analyses was determined using jModelTest 2.0 (Posada 2008). The GTR+I+G model with inverse gamma rate were selected for individual data from each partition with the combined aligned dataset. The phylogenetic tree was visualized in FigTree v. 1.4 (Rambaut 2012) and the layout of the tree was done in Adobe Illustrator v. CS5.1. The alignment of phylogenetic analyses was deposited in TreeBASE (https://www.treebase.org, submission number 24051).

## Results

### Molecular phylogeny

Following the results of preliminary phylogenetic analysis of the initial alignment (data not shown), the phylogenetic reconstruction of the second alignment was performed including SSU, LSU, and *tef*1sequences from 53 strains representing 10 different orders in the Dothideomycetes and one order in the Arthoniomycetes (Table 1). The three-gene dataset comprised of LSU sequences for all 52 ingroup sequences, 50 SSU sequences, and 36 *tef*1 sequences. After exclusion of ambiguous regions and introns, the combined dataset included 2555 characters (826 for LSU, 1012 for SSU, and 717 for *tef*1). In the BI analysis, the alignment has 952 distinct patterns, 600 parsimony-informative, 205 singleton sites, and 1750 constant sites.

**Figure 1.**
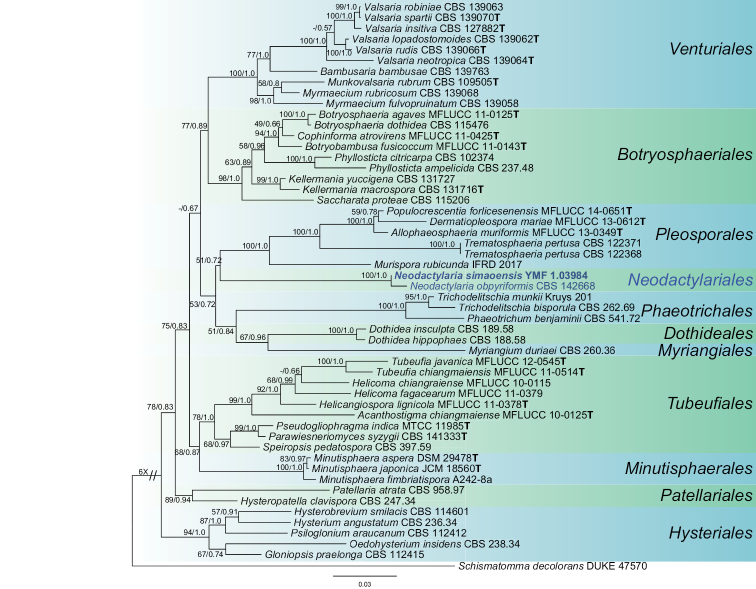
Maximum likelihood (RAxML) tree obtained by phylogenetic analyses of the combined LSU, SSU, and *tef*1 sequence alignment of 53 taxa belonging to the 11 orders shown to the right of the tree. The numbers of nodes in clades represent Maximum likelihood bootstrap support values (ML-BS, 0–100) and Bayesian posterior probabilities (BPP, 0–1.0). ML-BS greater than 50% and BPP above 0.5 are indicated at the nodes (ML-BS/BPP). The scalebar represents the number of changes. *Schismatomma decolorans*DUKE 47570 was used as outgroup. The strain numbers are noted after the species names with ex-type strains indicated with ^T^. The proposed new order is in boldface.

The best tree (RAxML) obtained using the ML analysis is shown as Fig. 1, with the support values from the ML and BI analyses plotted at the nodes. In this tree, our newly proposed species and *N. obpyriformis* formed a distinct clade within Dothideomycetes with significant ML bootstrap support (100%) and Bayesian sposterior probability (1.0). Moverover, the *Neodactylaria* clade is sister to the Pleosporales clade, but only with low bootstrap support values (51%) and Bayesian posterior probabilities (0.72). The results suggested that our strain belongs to the genus *Neodactylaria*. The order Pleosporales has characters that are very different from those of species of *Neodactylaria* and, therefore, we introduce a new order and new family, Neodactylariales and Neodactylariaceae, respectively, for this group of fungi. In addition, combined with morphological differences, our strain was described and illustrated herein as a new species of *Neodactylaria*.

### Taxonomy

#### Neodactylariales

Taxon classificationFungiNeodactylariales

H. Zheng & Z.F. Yu ord. nov.

AF15044A-545D-5989-A13A-DC22845D0F6A

MycoBank No: 830161

##### Type family.

Neodactylariaceae H. Zheng & Z.F. Yu.

##### Description.

Asexual morph from human-associated organs or saprobic on plant debris. Conidiophores acroauxic, macronematous, mononematous, branched or unbranched. Conidiogenous cells mono- and polyblastic, sympodially extended. Conidia solitary, hyaline or pale pigmented, smooth, verrucous or echinulate. Sexual morph not observed.

#### Neodactylariaceae

Taxon classificationFungiNeodactylarialesNeodactylariaceae

H. Zheng & Z.F. Yu fam. nov.

AB68F12F-5B57-5FF5-A3D2-61308C941F74

MycoBank No: 830162

##### Type genus.

*Neodactylaria* Guevara-Suarez, Deanna A. Sutton, Wiederh. & Gené.

##### Description.

Mycelium superficial or immersed, composed of branched, septate, hyaline to subhyaline hyphae. Conidiophores macronematous, mononematous, straight or flexuous, septate, unbranched. Conidiogenous cells terminal or intercalary, polyblastic, sympodial, with short-cylindrical denticles. Conidial secession schizolytic. Conidia solitary, smooth or finely echinulate. Sexual morph not observed.

#### Neodactylaria

Taxon classificationFungiNeodactylarialesNeodactylariaceae

Guevara-Suarez, Deanna A. Sutton, Wiederh. & Gené, in Crous et al. Persoonia 38: 345 (2017)

BF291FBD-1FE1-58B6-A48E-3AB23D4FDB58

##### Type species.

*Neodactylaria obpyriformis* Guevara-Suarez, Deanna A. Sutton, Wiederh. & Gené.

##### Description.

Mycelium superficial or immersed, composed of branched, septate, smooth-walled, hyaline to subhyaline hyphae. Conidiophores macronematous, mononematous, straight or flexuous, septate, unbranched, smooth-walled, pale to mid-brown. Conidiogenous cells polyblastic, sympodial extended, integrated, terminal or intercalary, denticulate, with short cylindrical denticles, pale to medium-brown. Conidial secession schizolytic. Conidia obpyriform to obclavate, unicellular or septate, attenuate, subulate or rostrate toward the obtuse apex, with a tiny, protuberant basal hilum, smooth or finely echinulate, subhyaline or pale brown. Sexual morph not observed.

#### Neodactylaria simaoensis

Taxon classificationFungiNeodactylarialesNeodactylariaceae

, H. Zheng & Z.F. Yu sp. nov.

C3BB9382-5F87-5597-B033-E76F1E1AD430

MycoBank No: 830160

Fig. 2

##### Diagnosis.

It is characterised by straight or flexuous, 2–4-septate, unbranched conidiophores, with denticulate conidiogenous cells and obclavate to long obpyriform, subulate or slightly rostrate towards the obtuse or rounded apex and 1–2 (–3)-septate conidia. Differs from *N. obpyriformis* by longer and slightly wider conidia and more septa.

##### Type.

China, Yunnan Province, Simao country, 100°59'19"N, 22°46'38"E, ca 1330 m alt., from submerged unidentified dicotyledonous leaves, 28 Oct 2013, Z.F. Yu, live culture YMF 1.03984 – ***holotype***, dried slide YMFT 1.03984.

##### Description.

Mycelium partly superficial or partly immersed, composed of branched, septate, hyaline to subhyaline, creeping, 1.0–2.0 μm wide hyphae. Conidiophores macronematous, mononematous, straight or flexuous, slightly geniculate towards the apex, 2–4-septate, unbranched, hyaline or pale brown, 38–86 (–129) × 3–4 μm, arising from the creeping hyphae pale brown. Conidiogenous cells polyblastic, indeterminate, sympodial extended, integrated, terminal or intercalary, denticulate with protuberant cylindrical denticles. Conidia solitary, obclavate to long obpyriform, subulate or slightly rostrate towards the obtuse or rounded apex, lumina micro-guttulate, 1–2 (–3)-septate, constricted at the septa, pale to mid brown, 15–40 × 3.6–6.5 μm, with a subhyaline, protuberant basal hilum up to 1 μm long.

##### Culture characteristics.

Colonies attaining 1 cm in diameter on CMA after 7 days at 25 °C. On CMA, colonies flat, floccose at the centre, lacking aerial mycelium towards periphery, white to cream-coloured, reverse same colour, sporulation abundant. On PDA, colonies flat, white to cream-coloured, margin entire; sporulation sparse.

##### Habitat and distribution.

In submerged dicotyledonous leaves from south-western China.

##### Teleomorph.

Not known.

##### Etymology.

The species epithet indicates its occurrence in the county of Simao, China.

##### Notes.

Based on a Blast search of NCBIs GenBank nucleotide database, the closest hits using the ITS sequences of *N. simaoensis* (GenBank MH379209) is *N. obpyriformis* (GenBank NR_154267, Identities = 545 / 569(96%), Gaps = 4 / 569(0%)). Morphologically, the new species, *N. simaoensis*, shares several characters with *N. obpyriformis* (type species): both have white to cream-coloured colonies, with short-cylindrical denticles as conidiogenous cells and obpyriform to slightly rostrate conidia (Crous et al. 2017). However, *N. simaoensis* differs from *N. obpyriformis* by having obviously longer and slightly wider conidia (15–40 × 3.6–6.5 μm vs 10–14 × 3–5 μm) and more septa.

**Figure 2.**
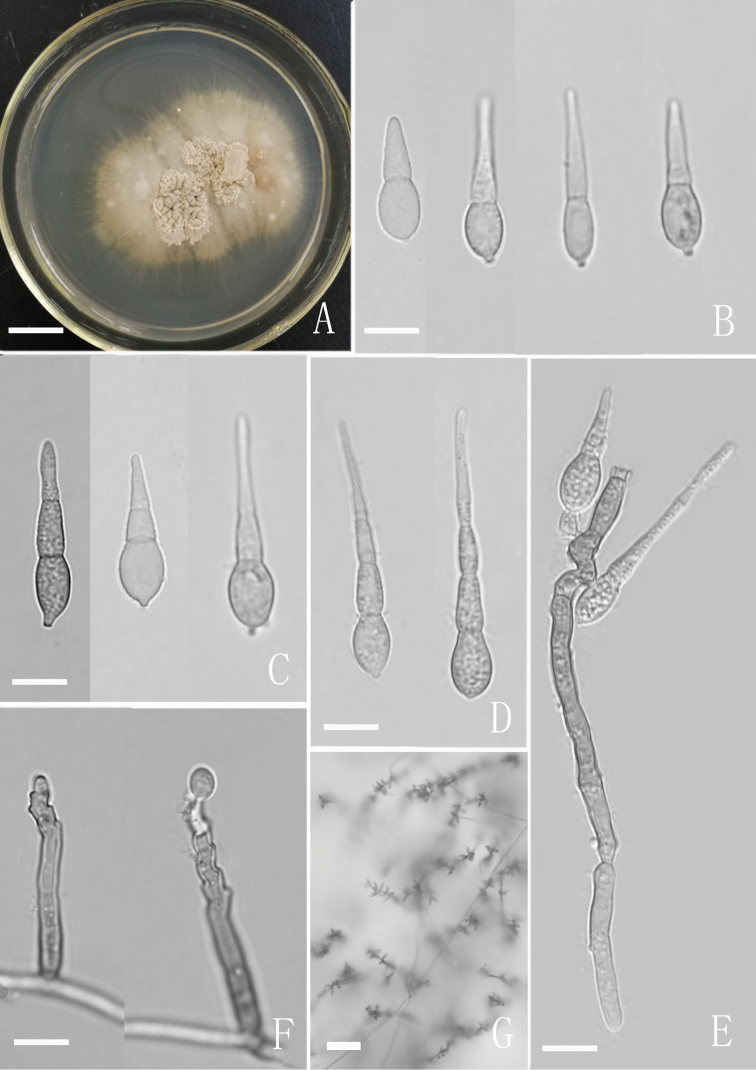
Culture and anamorph of *Neodactylaria simaoensis* (YMF 1.03984) **A** culture on CMA **B–D** conidia **E** conidia and conidiophores **F** immature conidium and conidiogenous cells **G** conidiophores and conidia under low power microscope. Scale bars: 1 cm (**A**); 10 μm (**B–F**); 50 μm (**G**).

## Discussion

Aquatic hyphomycetes, which have always been important members of the Dothideomycetes, play critical roles in the decomposition of organic compounds and nutrient cycling in aquatic habitats. Since Ingold (1942, 1943) first reported aquatic hyphomycetes in the 1940s, research on this group have been steadily increasing throughout the world. It was estimated that over 300 species of over 80 genera of aquatic hyphomycetes are reported worldwide (Kirk et al. 2008; Guo et al. 2015). Studies of aquatic hyphomycetes have revealed a huge fungal diversity. Our study again underlined the importance of these microorganisms for fungal taxonomic discovery.

In this study, a preliminary phylogenetic analysis combined SSU, LSU, *rpb*2, and *tef*1sequences from 74 representative taxa of Dothideomycetes and Arthoniomycetes revealed the *Neodactylaria* as a unique clade within Dothideomycetes (data not shown). The second phylogenetic analyses using three loci (SSU, LSU, *tef*1) also showed our new collected strain and *N. obpyriformis* form a strongly supported monophyletic and distinct clade (ML-BS = 100%, BPP = 1.0) within the Dothideomycetes (Fig. 1). In this tree, the *Neodactylaria* clade is close to the *Pleosporales* but with low support (ML-BS = 51%, BPP = 0.72). The original study on *N. obpyriformis*, which conducted a phylogenetic analysis of the LSU sequence, also showed that *Neodactylaria* is related to Dothideomycetes, but with an uncertain taxonomic position at the ordinal level and family level (Crous et al. 2017). Thus, we establish a new order (Neodactylariales) and family (Neodactylariaceae) within the Dothideomycetes for this unique clade.

The genus *Neodactylaria* is morphologically similar to two species of the genus *Dactylaria*, *D. kumamotoensis* and *D. madresensis*, which were described by Matsushima from soil and plant debris in Japan and India, respectively (Matsushima 1981, 1984). Although these two fungi in *Dactylaria* could be congeneric with *N. simaoensis*, they are only known from the type collection and no living cultures are available for molecular comparison. Morphologically, the conidia of *N. simaoensis* are smaller than *D. kumamotoensis* and are distinguished from *D. madresensis* by their size and the number of septa. In addition, the genus *Dactylaria* is heterogeneous. Related information showed that the classification position of *D. kumamotoensis* was in the order Helotiales, the class Leotiomycetes (http://www.indexfungorum.org/Names/NamesRecord.asp?RecordID=111390), but most *Dactylaria* species were placed in the Sordariomycetes (Crous et al. 2017). Thus, although the genus *Neodactylaria* shares some morphological characters with the genus *Dactylaria*, *Neodactylaria* was placed in the Dothideomycetes by phylogenetical analysis and was phylogenetically distant from *Dactylaria*.

In the Dothideomycetes, many orders show various morphological characteristics and lifestyles, such as the order Pleosporales. In our new order, the two species within genus *Neodactylaria* also have different habitats: *N. obpyriformis* was found from human bronchoalveolar lavage in the USA, but *N. simaoensis* was found from submerged decaying leaves in China. Therefore, it seems fungi in this genus may be broadly distributed in different habitats.

The class Dothideomycetes is one of the most important and diverse classes in the phylum Ascomycota. It comprises pathogenic fungi, aquatic hyphomycetes, fungi with different life cycles and habitats, and also fungi with biotechnological potential (Wijayawardene et al. 2014; Santos et al. 2015; Woudenberg et al. 2015; Zheng et al. 2020b). In recent years, this class has received significant attention, and several papers have highlighted its importance to fungal taxonomy, based on its fungal diversity and on new studies performed to improve the classification of dothideomycetous fungi (Schoch et al. 2009a; Hyde et al. 2013; Wijayawardene et al. 2014). In Dothideomycetes, most families comprise both sexual genera and asexual genera and only a few families are totally comprised of asexual genera, such as Cladosporiaceae Nann., which contains seven asexual hyphomycetous genera and Neodevriesiaceae Quaedvlieg & Crous, which contains one asexual hyphomycetous genus (Wijayawardene et al. 2014). However, the order Lichenoconiales, only comprising one family, was also established with an asexual genus (Hyde et al. 2013). Here, we added a new order containing only an asexual genus to Dothideomycetes. These results show asexual genera have equal status to sexual genera at various taxon ranks. In addition, the description of Neodactylariales, as a new order in this study, highlights the need to collect fungal biodiversity from a range of diverse environments and substrates, as these diverse niches frequently harbour fungal lineages that are still missing in current phylogenetic studies.

## Supplementary Material

XML Treatment for Neodactylariales

XML Treatment for Neodactylariaceae

XML Treatment for Neodactylaria

XML Treatment for Neodactylaria simaoensis

## References

[B1] Cortinas MN Burgess T Dell B Xu D Crous PW Wingfield BD Wingfield MJ (2006) First record of *Colletogloeopsis zuluense* comb. nov., causing stem canker of *Eucalyptus* in China. Mycological Research 110: 229–236. 10.1016/j.mycres.2005.08.012 16378717

[B2] Crous PW Braun U Groenewald JZ (2007) *Mycosphaerella* is polyphyletic. Studies in Mycology 58: 1–32. 10.3114/sim.2007.58.01 18490994 PMC2104738

[B3] Crous PW Wingﬁeld MJ Burgess TI Hardy GESTJ Barber PA Alvarado P Barnes CW Buchanan PK Heykoop M Moreno G Thangavel R van der Spuy S Barili A Barrett S Cacciola SO Cano-Lira JF Crane C Decock C Gibertoni TB Guarro J Guevara-Suarez M Hubka V Kolařík M Lira CRS Ordoñez ME Padamsee M Ryvarden L Soares AM Stchigel AM Sutton DA Vizzini A Weir BS Acharya K Aloi F Baseia IG Blanchette RA Bordallo JJ Bratek Z Butler T Cano-Canals J Carlavilla JR Chander J Cheewangkoon R Cruz RHSF da M Dutta AK Ercole E Escobio V Esteve-Raventós F Flores JA Gené J Góis JS Haines L Held BW Jung MH Hosaka K Jung T Jurjević Ž Kautman V Kautmanova I Kiyashko AA Kozanek M Kubátová A Lafourcade M La Spada F Latha KPD Madrid H Malysheva EF Manimohan P Manjón JL Martín MP Mata M Merényi Z Morte A Nagy I Normand AC Paloi S Pattison N Pawłowska J Pereira OL Petterson ME Picillo B Raj KNA Roberts A Rodríguez A Rodríguez-Campo FJ Romański M Ruszkiewicz-Michalska M Scanu B Schena L Semelbauer M Sharma R Shouche YS Silva V Staniaszek-Kik M Stielow JB Tapia C Taylor PWJ Toome-Heller M Vabeikhokhei JMC van Diepeningen AD Van Hoa N Van Tri M Wiederhold NP Wrzosek M Zothanzama J Groenewald JZ (2017) Fungal Planet description sheets: 558–624. Persoonia 38: 240–384. 10.3767/003158517X698941 29151634 PMC5645186

[B4] Da Cunha KC Sutton DA Fothergill AW Cano J Gené J Madrid H De Hoog S Crous PW Guarro J (2012) Diversity of *Bipolaris* species in clinical samples in the United States and their antifungal susceptibility profiles. Journal of Clinical Microbiology 50: 4061–4066. 10.1128/JCM.01965-12 23052310 PMC3502984

[B5] Da Cunha KC Sutton DA Fothergill AW Gené J Cano J Madrid H De Hoog S Crous PW Guarro J (2013) In vitro antifungal susceptibility and molecular identity of 99 clinical isolates of the opportunistic fungal genus *Curvularia*. Diagnostic Microbiology and Infectious Disease 76: 168–174. 10.1016/j.diagmicrobio.2013.02.034 23558007

[B6] Damm U Verkley GJM Crous PW Fourie PH Haegi A Riccioni L (2008) Novel *Paraconiothyrium* species on stone fruit trees and other woody hosts. Persoonia 20: 9–17. 10.3767/003158508X286842 20467483 PMC2865355

[B7] De Wit PJGM Van der Burgt A Ökmen B Stergiopoulos I Abd-Elsalam KA Aerts AL Bahkali AH Beenen HG Chettri P Cox MP Datema E De Vries RP Dhillon B Ganley AR Griffiths SA Guo Y Hamelin RC Henrissat B Kabir MS Jashni MK Kema G Klaubauf S Lapidus A Levasseur A Lindquist E Mehrabi R Ohm RA Owen TJ Salamov A Schwelm A Schijlen E Sun H Van den Burg HA Van Ham RCHJ Zhang S Goodwin SB Grigoriev IV Collemare J Bradshaw RE (2012) The genomes of the fungal plant pathogens *Cladosporium fulvum* and *Dothistroma septosporum* reveal adaptation to different hosts and lifestyles but also signatures of common ancestry. PLOS Genetics 8(11): 1–22. 10.1371/journal.pgen.1003088 PMC3510045 23209441

[B8] Don R Cox P Wainwright B Baker K Mattick J (1991) ‘Touchdown’ PCR to circumvent spurious priming during gene amplification. Nucleic Acids Research 19: 4008. 10.1093/nar/19.14.4008 PMC328507 1861999

[B9] Gams W Hoekstra ES Aptroot A (1998) CBS Course of Mycology, Fourth Edition. Centraalbureau voor Schimmelcultures, Baarn.

[B10] Guo MT Qian WY Li JY Yu ZF (2015) One genus and three species of aquatic hyphomycetes new to china. Mycosystema 2015(6): 1205–1208.

[B11] Guo JS Zhang Z Qiao M Yu ZF (2019) *Phalangispora sinensis* sp. nov. from Yunnan, China and two new members of *Wiesneriomycetaceae*. International Journal of Systematic and Evolutionary Microbiology 69(10): 3207–3213. 10.1099/ijsem.0.003612 31339482

[B12] Hall TA (1999) BioEdit: a user-friendly biological sequence alignment editor and analysis program for Windows 95/98/NT. Nucleic Acids Symposium Series 41: 95–98.

[B13] Hernandez-Restrepo M Gené J Castaneda-Ruiz RF Mena-Portales J Crous PW Guarro J (2017) Phylogeny of saprobic microfungi from Southern Europe. Studies in Mycology 86: 53–97. 10.1016/j.simyco.2017.05.002 28626275 PMC5470572

[B14] Hirayama K Tanaka K Raja HA Miller AN Shearer CA (2010) A molecular phylogenetic assessment of *Massarina ingoldiana* sensu lato. Mycologia 102: 729–746. 10.3852/09-230 20524604

[B15] Hyde KD Jones EBG Liu JK Ariyawansa HA Zhang M (2013) Families of Dothideomycetes. Fungal Diversity 63: 1–313. 10.1007/s13225-013-0263-4

[B16] Hyde KD Nilsson RH Alias SA Ariyawansa HA Blair JE Cai L De Cock AWAM Dissanayake AJ Glockling SL Goonasekara ID Gorczak M Hahn M Jayawardena RS Van Kan JAL Laurence MH Lévesque CA Li XH Liu JK Maharachchikumbura SSN Manamgoda DS Martin FN McKenzie EHC McTaggart AR Mortimer PE Nair PVR Pawłowska J Rintoul TL Shivas RG Spies CFJ Summerell BA Taylor PWJ Terhem RB Udayanga D Vaghefi N Walther G Wilk M Wrzosek M Xu JX Yan JY Zhou N (2014) One stop shop: backbones trees for important phytopathogenic genera: I. Fungal Diversity 67: 21–125. 10.1007/s13225-014-0298-1

[B17] Inderbitzin P Landvik S Abdel-Wahab MA Berbee ML (2001) *Aliquandostipitaceae*, a new family for two new tropical ascomycetes with unusually wide hyphae and dimorphic ascomata. American Journal of Botany 88: 52–61. 10.2307/2657126 11159126

[B18] Ingold CT (1942) Aquatic Hyphomycetes of decaying alder leaves. Transactions of the British Mycological Society 25: 339–417. 10.1016/S0007-1536(42)80001-7

[B19] Ingold CT (1943) Further observations on aquatic Hyphomycetes. Transactions of the British Mycological Society 26: 104–115. 10.1016/S0007-1536(43)80015-2

[B20] Jiang SH Hawksworth DL Lücking R Wei JC (2020) A new genus and species of foliicolous lichen in a new family of Strigulales (Ascomycota: Dothideomycetes) reveals remarkable class-level homoplasy. IMA Fungus 11: 1–1. 10.1186/s43008-019-0026-2 32617253 PMC7325298

[B21] Katoh K Standley DM (2013) MAFFT multiple sequence alignment software version 7: improvements in performance and usability. Molecular Biology and Evolution 30: 772–780. 10.1093/molbev/mst010 23329690 PMC3603318

[B22] Kindermann J El-Ayouti Y Samuels GJ Kubicek CP (1998) Phylogeny of the genus *Trichoderma* based on sequence analysis of the internal transcribed spacer region 1 of the rDNA clade. Fungal Genetics and Biology 24: 298–309. 10.1006/fgbi.1998.1049 9756711

[B23] Kirk PM Cannon PF Minter DW Stalpers JA (2008) Ainsworth & Bisby’s Dictionary of the Fungi, Tenth Edition. CABI, Wallingford. 10.1079/9780851998268.0000

[B24] Liu JK Hyde KD Jeewon R Phillips AJL Maharachchikumbura SSN Ryberg M Liu ZY Zhao Q (2017) Ranking higher taxa using divergence times: a case study in Dothideomycetes. Fungal Diversity 84: 75–99. 10.1007/s13225-017-0385-1

[B25] Liu YJ Whelen S Hall BD (1999) Phylogenetic relationships among ascomycetes: evidence from an RNA polymerase II subunit. Molecular Biology and Evolution 16: 1799–1808. 10.1093/oxfordjournals.molbev.a026092 10605121

[B26] Lynne B (2016) Genetics – variation, sexuality, and evolution. In: Watkinson SC Boddy L Money NP (Eds) The Fungi, Third Edition.Academic Press, London, 99–139. 10.1016/B978-0-12-382034-1.00004-9

[B27] Manamgoda DS Cai L McKenzie EHC Crous PW Madrid H Chukeatirote E Shivas RG Tan YP Hyde KD (2012) A phylogenetic and taxonomic re-evaluation of the *Bipolaris*-*Cochliobolus*-*Curvularia* complex. Fungal Diversity 56: 131–144. 10.1007/s13225-012-0189-2

[B28] Mapook A Hyde KD Dai D Li J Jones EBG Bahkali AH Boonmee S (2016) Muyocopronales, ord. nov., (Dothideomycetes, Ascomycota) and a reappraisal of *Muyocopron* species from northern Thailand. Phytotaxa 265(3): 225–237. 10.11646/phytotaxa.265.3.3

[B29] Matsushima T (1981) Matsushima Mycological Memoirs 2. Matsushima Fungus Collection, Kobe, 68 pp.

[B30] Matsushima T (1984) Matsushima Mycological Memoirs No.3: Mycologia 76(2): 1–90. 10.2307/3793127

[B31] Mel’nik VA Castaneda-Ruiz RF Granados M (2013) A new species of *Heliocephala* from Vietnam. Mycotaxon 123: 281–284. 10.5248/123.281

[B32] Myers N Mittermeier RA Mittermeier CG da Fonseca GA Kent J (2000) Biodiversity hotspots for conservation priorities. Nature 403: 853–858. 10.1038/35002501 10706275

[B33] Nieuwenhuijzen VE Miadlikowska J Houbraken J Adan OO Lutzoni F Samson RA (2016) Wood staining fungi revealed taxonomic novelties in Pezizomycotina: New order Superstratomycetales and new species *Cyanodermella oleoligni*. Studies in Mycology 85: 107–124. 10.1016/j.simyco.2016.11.008 28050056 PMC5198870

[B34] Nilsson RH Larsson K-H Taylor AFS Bengtsson-Palme J Jeppesen TS Schigel D Kennedy P Picard K Glöckner FO Tedersoo L Saar I Kõljalg U Abarenkov K (2019) The UNITE database for molecular identification of fungi: handling dark taxa and parallel taxonomic classifications. Nucleic Acids Research 47: 259–264. 10.1093/nar/gky1022 PMC6324048 30371820

[B35] Ohm RA Feau N Henrissat B Schoch CL Horwitz BA Barry KW Condon BJ Copeland AC Dhillon B Glaser F Hesse CN Kosti I LaButti K Lindquist EA Lucas S Salamov AA Bradshaw RE Ciuffetti L Hamelin RC Kema GHJ Lawrence C Scott JA Spatafora JW Turgeon BG De Wit PJGM Zhong S Goodwin SB Grigoriev IV (2012) Diverse life styles and strategies of plant pathogenesis encoded in the genomes of eighteen DothideomycetesFungi PLoS Pathogens 8(12): e1003037. 10.1371/journal.ppat.1003037 PMC3516569 23236275

[B36] Qiao M Tian WG Castañeda-Ruiz RF Xu JP Yu ZF (2019a) Two new species of *Verruconis* from Hainan, China. MycoKeys 48: 41–53. 10.3897/mycokeys.48.32147 30872943 PMC6414473

[B37] Qiao M Zheng H Zhang Z Yu ZF (2019b) *Seychellomyces sinensis* sp. nov. from China. Mycotaxon 134(2): 391–398. 10.5248/134.391

[B38] Posada D (2008) jModelTest: phylogenetic model averaging. Molecular Biology and Evolution 25: 1253–1256. 10.1093/molbev/msn083 18397919

[B39] Rambaut A (2012) FigTree v1.4.2. http://tree.bio.ed.ac.uk/software/figtree/

[B40] Rao V Reddy KA Hoog GSD (1984) *Heliocephala*, a new genus of dematiaceous Hyphomycetes. Persoonia 12: 239–242.

[B41] Rayner RW (1970) A Mycological Colour Chart. Commonwealth Mycological Institute and British Mycological Society, Kew.

[B42] Santos MGS Bezerra JDP Svedese VM Sousa MA Silva DCV Maciel MHC Paiva LM Porto ALF Souza-Motta CM (2015) Screening of endophytic fungi from cactus of the Brazilian tropical dry forest according to their L-asparaginase activity. Sydowia 67: 147–156.

[B43] Sayers EW Cavanaugh M Clark K Ostell J Pruitt KD Karsch-Mizrachi I (2019) GenBank. Nucleic Acids Research 47: 94–99. 10.1093/nar/gky989 PMC6323954 30365038

[B44] Schoch CL Crous PW Groenewald JZ Boehm EW Burgess TI De Gruyter J De Hoog GS Dixon LJ Grube M Gueidan C Harada Y Hatakeyama Hirayama SK Hosoya T Huhndorf SM Hyde KD Jones EBG Kohlmeyer J Kruys A Li YM Lücking R Lumbsch HT Marvanová L Mbatchou JS McVay AH Miller AN Mugambi GK Muggia L Nelsen MP Nelson P Owensby CA Phillips AJL Phongpaichit S Pointing SB Pujade-Renaud V Raja HA Plata ER Robbertse B Ruibal C Sakayaroj J Sano T Selbmann L Shearer CA Shirouzu T Slippers B Suetrong S Tanaka K Volkmann-Kohlmeyer B Wingfield MJ Wood AR Woudenberg JHC Yonezawa H Zhang Y Spatafora JW (2009b) A class-wide phylogenetic assessment of Dothideomycetes. Studies in Mycology 64: 1–15. 10.3114/sim.2009.64.01 20169021 PMC2816964

[B45] Schoch CL Sung GH Lopez-Giraldez F Townsend JP Miadlikowska J Hofstetter V Robbertse B Matheny PB Kauff F Wang Z Gueidan C Andrie RM Trippe K Ciufetti LM Wynns A Fraker E Hodkinson BP Bonito G Groenewald JZ Arzanlou M De Hoog GS Crous PW Hewitt D Pfister DH Peterson K Gryzenhout M Wingfield MJ Aptroot A Suh SO Blackwell M Hillis DM Griffith GW Castlebury LA Rossman AY Lumbsch HT Lücking R Büdel B; Rauhut A Diederich P Ertz D Geiser DM Hosaka K Inderbitzin P Kohlmeyer J Volkmann-Kohlmeyer B Mostert L O’Donnell K Sipman H Rogers JD Shoemaker RA Sugiyama J Summerbell RC Untereiner W Johnston PR Stenroos S Zuccaro A Dyer PS Crittenden PD Cole MS Hansen K Trappe JM Yahr R Lutzoni F Spatafora JW (2009a) The Ascomycota tree of life: a phylum-wide phylogeny clarifies the origin and evolution of fundamental reproductive and ecological traits. Systematic Biology 58: 224–239. 10.1093/sysbio/syp020 20525580

[B46] Siu K Lzumi AK (2004) Phaeohyphomycosis caused by *Coniothyrium*. Cutis 73: 127–130. 15027518

[B47] Stergiopoulos I Kourmpetis YAI Slot JC Bakker FT De Wit JPGM Rokas A (2012) In silico characterization and molecular evolutionary analysis of a novel superfamily of fungal effector proteins. Molecular Biology and Evolution 29: 3371–3384. 10.1093/molbev/mss143 22628532

[B48] Suetrong S Boonyuen N Pang K Ueapattanakit J Klaysuban A Sri-indrasutdhi V Sivichai S Jones EBG (2011) A taxonomic revision and phylogenetic reconstruction of the Jahnulales (Dothideomycetes), and the new family Manglicolaceae. Fungal Diversity 51: 163–188. 10.1007/s13225-011-0138-5

[B49] Turner D Kovacs W Kuhls K Lieckfeldt E Peter B Arisan-Atac I Strauss J Samuels GJ Börner T Kubicek CP (1997) Biogeography and phenotypic variation in Trichoderma sect. Longibrachiatum and associated *Hypocrea* species. Mycological Research 101: 449–459. 10.1017/S0953756296002845

[B50] Verkley GJM da Silva M Wicklow DT Crous PW (2004) *Paraconiothyrium*, a new genus to accommodate the mycoparasite *Coniothyrium minitans*, anamorphs of *Paraphaeosphaeria*, and four new species. Studies in Mycology 50: 323–335.

[B51] Vilgalys R Hester M (1990) Rapid genetic identification and mapping of enzymatically amplified ribosomal DNA from several *Cryptococcus* species. Journal of Bacteriology 172: 4238–4246. 10.1128/JB.172.8.4238-4246.1990 2376561 PMC213247

[B52] Voglmayr H Gardiennet A Jaklitsch WM (2016) *Asterodiscus* and *Stigmatodiscus*, two new apothecial Dothideomycete genera and the new order Stigmatodiscales. Fungal Diversity 80(1): 271–284. 10.1007/s13225-016-0356-y 27818618 PMC5075026

[B53] White TJ Bruns T Lee S Taylor J (1990) Amplification and direct sequencing of fungal ribosomal RNA genes for phylogenetics. PCR Protocols: a guide to methods and applications 18: 315–322. 10.1016/B978-0-12-372180-8.50042-1

[B54] Wijayawardene NN Crous PW Kirk PM Hawksworth DL Boonmee S Braun U Dai DQ D’souza MJ Diederich P Dissanayake A Doilom M Hongsanan S Jones EBG Groenewald JZ Ruvishika Jayawardena R Lawrey JD Liu JK Lücking R Madrid H Manamgoda DS Muggia L Nelsen MP Phookamsak R Suetrong S Tanaka K Thambugala KM Wanasinghe DN Wikee S Zhang Y Aptroot A Ariyawansa HA Bahkali AH Bhat DJ Gueidan C Chomnunti P De Hoog GS Knudsen K Li WJ McKenzie EHC Miller AN Phillips AJL Piątek M Raja HA Shivas RS Slippers B Taylor JE Tian Q Wang Y Woudenberg JHC Cai L Jaklitsch WM Hyde KD (2014) Naming and outline of Dothideomycetes – 2014 including proposals for the protection or suppression of generic names. Fungal Diversity 69: 1–55. 10.1007/s13225-014-0309-2 27284275 PMC4896388

[B55] Wijayawardene NN Hyde KD Lumbsch HT Liu JK Maharachchikumbura SSN Ekanayaka AH Tian Q Phookamsak R (2018) Outline of Ascomycota: 2017. Fungal Diversity 88: 167–263. 10.1007/s13225-018-0394-8

[B56] Wikee S Lombard L Crous PW Nakashima C Motohshi K Chukeatirote E Alias SA McKenzie EHC Hyde KD (2013a) *Phyllosticta capitalensis*, a widespread endophyte of plants. Fungal Diversity 60: 91–105. 10.1007/s13225-013-0235-8

[B57] Wikee S Lombard L Nakashima C Motohashi K Chukeatirote E Alias SA McKenzie EHC Hyde KD (2013b) A phylogenetic re-evaluation of *Phyllosticta* (Botryosphaeriales). Studies in Mycology 76: 1–29. 10.3114/sim0019 24302788 PMC3825230

[B58] Wikee S Udayanga D Crous PW Chukeatirote E Mckenzie EHC Bahkali AH Dai DQ Hyde KD (2011) *Phyllosticta* – an overview of current status of species recognition. Fungal Diversity 51: 43–61. 10.1007/s13225-011-0146-5

[B59] Woudenberg JHC Seidl MF Groenewald JZ de Vries M Stielow JB Thomma BPHJ Crous PW (2015) Alternaria section Alternaria: Species, formae speciales or pathotypes? Studies in Mycology 82: 1–21. 10.1016/j.simyco.2015.07.001 PMC4774270 26951037

[B60] Yu ZF Lv YF Feng B Qiao M (2019) *Lemonniera yulongensis* sp. nov. from Yunnan, China. Mycotaxon 134(1): 177–181. 10.5248/134.177

[B61] Zhang Y Qiao M Xu JP Baral HO Zhang KQ Yu ZF (2020) Morphological and molecular characterization of two new species of *Orbilia* (Orbiliomycetes) from China. International Journal of Systematic and Evolutionary Microbiology 70: 2664–2676. 10.1099/ijsem.0.004088 32238230

[B62] Zheng H Yang XQ Xu JP Yu ZF (2020a) *Beltrania sinensis* sp. nov., a new endophytic fungus from China and a key to species of the genus. International Journal of Systematic and Evolutionary Microbiology 70(2): 1178–1185. 10.1099/ijsem.0.003897 31860431

[B63] Zheng H Yu ZF Xu JP Castañeda-Ruiz RF Qiao M (2020b) *Ramichloridium endophyticum* sp. nov., a new species of endophytic fungi from *Potamogeton pectinatus* in Tibet, China. International Journal of Systematic and Evolutionary Microbiology 70: 3433–439. 10.1099/ijsem.0.004190 32375982

[B64] Zheng H Zhang Z Liu DZ Yu ZF (2019) *Memnoniella sinenesis* sp. nov., a new species from China and a key to species of the genus. International Journal of Systematic and Evolutionary Microbiology 69(10): 3161–3169. 10.1099/ijsem.0.003605 31390326

